# 

**Published:** 1877-01

**Authors:** 


					﻿Vol. 24.	JANUARY, 1877.	No. 1.
TWENTY-FOURTH YEAR.
HALL’S
Journal of Health.
PUBLISHED AT 137 EIGHTH STREET, NEW YORK,
Four Doors Bast of 756 Broadway, f
. E. H. GIBBS, M.D., Editor.^
AGENTS TOR THE TRADE:	>
AMERICAN NEWS COMPANY, m'NASSAU STREET.
NEW YORK NEWS COMPANY, 18 BEEKMAN STREET.
Terms, $ 1.60 a Year.
SINGLE NUMBER, 16 CENTS.
John Exnt, Printer, 11 Frankfort St., New York.
				

